# On the Security of Bluetooth Low Energy in Two Consumer Wearable Heart Rate Monitors/Sensing Devices

**DOI:** 10.3390/s22030988

**Published:** 2022-01-27

**Authors:** Yeṣem Kurt Peker, Gabriel Bello, Alfredo J. Perez

**Affiliations:** TSYS School of Computer Science, Columbus State University, Columbus, GA 31907, USA; bello_gabriel@columbusstate.edu (G.B.); perez_alfredo@columbusstate.edu (A.J.P.)

**Keywords:** Bluetooth Smart, Bluetooth LE, security, privacy, wearables, fitness trackers, heart rate, BLE keyboards, usable privacy, usable security

## Abstract

Since its inception in 2013, Bluetooth Low Energy (BLE) has become the standard for short-distance wireless communication in many consumer devices, as well as special-purpose devices. In this study, we analyze the security features available in Bluetooth LE standards and evaluate the features implemented in two BLE wearable devices (a Fitbit heart rate wristband and a Polar heart rate chest wearable) and a BLE keyboard to explore which security features in the BLE standards are implemented in the devices. In this study, we used the ComProbe Bluetooth Protocol Analyzer, along with the ComProbe software to capture the BLE traffic of these three devices. We found that even though the standards provide security mechanisms, because the Bluetooth Special Interest Group does not require that manufacturers fully comply with the standards, some manufacturers fail to implement proper security mechanisms. The circumvention of security in Bluetooth devices could leak private data that could be exploited by rogue actors/hackers, thus creating security, privacy, and, possibly, safety issues for consumers and the public. We propose the design of a Bluetooth Security Facts Label (BSFL) to be included on a Bluetooth/BLE enabled device’s commercial packaging and conclude that there should be better mechanisms for informing users about the security and privacy provisions of the devices they acquire and use and to educate the public on protection of their privacy when buying a connected device.

## 1. Introduction

Bluetooth LE (Low Energy), otherwise known as Bluetooth Smart, has seen widespread adoption in various technological fields since its release in 2010. From Apple Watches to heart rate sensors, Bluetooth LE serves as a communication technology with a low energy consumption cost. Fitness trackers and wearables, an undoubtable niche in the Bluetooth LE community, have become commonplace [1]. Bluetooth LE is also used for occupancy tracking in office spaces [2], emergency management in buildings [3], smart energy management [4], and many other applications [5]. With billions of devices implementing Bluetooth LE technology (including smart phones and other wearables), questions of its security arise. Considering the type of data that can be transmitted between Low Energy devices, which can range from heartbeats to geolocation data, security and privacy concerns must be considered when acquiring a BLE device.

The Bluetooth Special Interest Group (Bluetooth SIG) is the standards organization that oversees the development of Bluetooth standards and its licensing to manufacturers. In 2010, Bluetooth SIG integrated LE with Bluetooth 4.0 Core Specification to include features to enable lower power consumption, lower complexity, and lower cost when compared to Basic Rate/Enhanced Data Rate (BR/EDR) [6]. That is, Bluetooth LE contrasts from other Bluetooth versions by reducing computational costs for devices. The design of the LE specifications allows room for manufacturing flexibility, but the core architecture of a device adheres to a standardized structure. The specifications include the ability for LE devices to implement many security features, including association models, encryption, authentication, and random addressing, which can be used to ensure the security and privacy of the data transmitted during a connection. Bluetooth SIG recommends but does not require security options to be implemented. The manufacturer can decide whether security measures are in place to protect consumers.

The first phone to implement the 4.0 specification was the iPhone 4S released in October 2011. Bluetooth SIG then released improvements, including the Bluetooth 4.1 Specification released in 2013, Bluetooth 4.2 Specification in 2014, and Bluetooth 5 in 2016. Bluetooth 4.1 had more efficient device power management compared to 4.0 and allows devices to act both as hubs and end points. It also introduces BR/EDR Secure Connections and LE Privacy 1.1. Bluetooth 4.2 increases speed and provides improved security for LE via Secure Connections for pairing. It also allows IPv6 connection option for Bluetooth LE devices [7]. Bluetooth 5 is a commonly used term to mean Bluetooth 5.0. It allows for much higher range and data transfer speeds and ability to transmit audio between two devices at the same time. Since the release of 5.0, Bluetooth SIG has released versions 5.1, 5.2, and 5.3 in 2019, 2020, and 2021, respectively. Version 5.1 improves connection speed and reliability, provides more accurate location detection, and introduces Randomized Advertising Channel Indexing for more efficient broadcasting. Bluetooth 5.2 features isochronous channels, allowing for multiple connections of Bluetooth devices to single sources, an enhanced attribute protocol, and LE power control [8]. Finally, Bluetooth 5.3 comes with various enhancements to improve reliability, energy efficiency, and user experience in many types of Bluetooth enabled products [9].

Even though most smart phones integrate the most recent LE technology in the latest releases, many other devices are not manufactured with the latest standards. Because the communication between two Bluetooth devices (according to Bluetooth standards) is determined by the oldest of the Bluetooth versions implemented between the devices attempting to communicate, a good portion of the Bluetooth LE communication today follows the 4.0 or 4.1 technology. In this study, we focus on BLE-enabled Bluetooth 4.0 and 4.1 devices. In particular, we investigate two heart rate wearable sensing devices (a Polar H7 heart rate monitor and Fitbit Charge 4) and a BLE Bluebyte keyboard. These devices are available for the public in the market today and are preferred by some consumers due to their affordable prices. These devices are advertised as Bluetooth LE, but their specifications do not include any information about the security features they implement.

We note that the consumer is rarely informed about the version of Bluetooth that is implemented in a device. For some devices, we determine the integrated version only after capturing the traffic, as the specifications that accompany the device did not indicate a version (except for stating that it is a Bluetooth LE device). This is a concern for consumer privacy, as different versions of Bluetooth provide different mechanisms for security. The contributions of this work are as follows:

- We report on the architecture and security features available in Bluetooth LE 4.0 and 4.1.
- We describe a testbed to study BLE implementations on hardware.
- We investigate security issues on implementations of Bluetooth LE in three commercially available devices, namely a Fitbit wristband device, a chest wearable, and a BLE keyboard. The devices are manufactured by popular brands, and to the best of our knowledge, the specific BLE manufacturers’ security implementations (the actual hardware/software BLE implementations done by the manufacturers) of these three devices have not been previously investigated.
- We propose the incorporation of a Bluetooth Security Facts Label (BSFL), which the Bluetooth Special Interest Group (SIG) and/or manufacturers could incorporate into the Bluetooth-enabled device’s commercial product packaging to help the consumer identify the security/privacy features of a device.

The rest of the paper is structured as follows: Section 2 provides an overview of the Bluetooth LE architecture relevant to our study and available security features in the architecture. It briefly describes previous studies on Bluetooth LE security. Section 3 describes the tools and methodology used in our study. Section 4 presents the results of our study, followed by a discussion of the results in Section 5. Finally, Section 6 concludes our work and provides directions for future research.

## 2. Bluetooth Low Energy Protocol and Security

In this section, we describe the basics of Bluetooth LE architecture and communication. For the discussions below (and otherwise specified), we will focus on BLE 4.0 and 4.1.

### 2.1. Bluetooth Low Energy Protocol Stack Architecture

**Figure 1:**
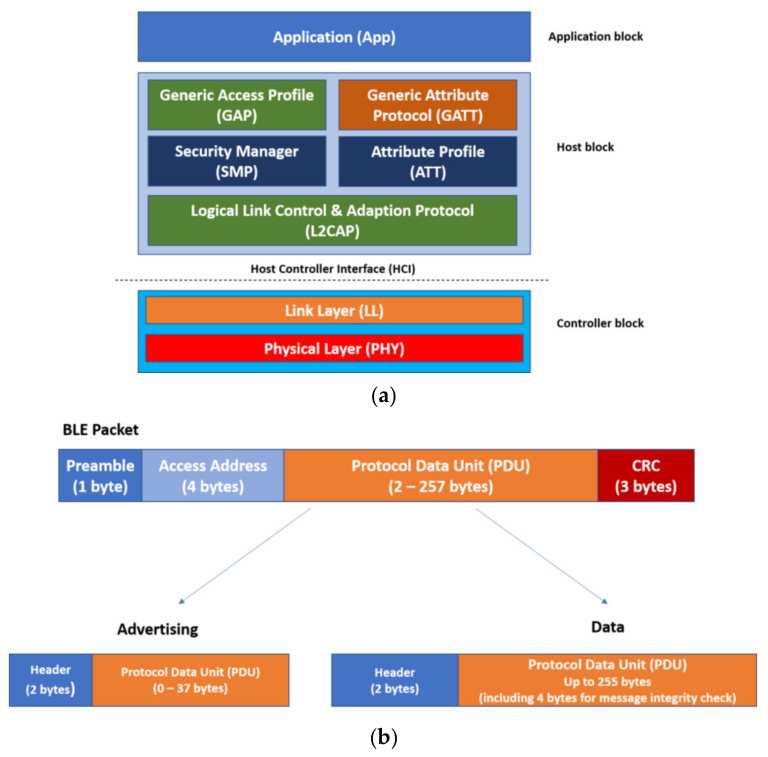
Bluetooth Low Energy (BLE) protocol stack and protocol packet format. (**a**) BLE Protocol stack; (**b**) BLE protocol packet format.

The BLE protocol stack architecture is composed of three blocks (as shown in Figure 1a):

1. Application block: The application block implements software based on the manufacturer’s need, which may vary from device to device.
2. Host block: This block is responsible for the protocols and profiles implemented in BLE devices and defines the packet semantics.
3. Controller block: This block features much of the device’s hardware, including the radio interface and its physical characteristics. This block is responsible for data broadcasts over the wireless media.

The BLE specification defines one type of packet with two different payloads to be transmitted by BLE devices. These payloads are advertising packets and data packets (Figure 1b). Advertising packets are used when a device is either in discovery mode or is broadcasting. On the other hand, data packets are only used when a device has established a connection with another device.

Both packets can potentially be used to transmit application data. However, data packets are allotted more payload space than advertising packets and, as a result, are used when more data needs to be transmitted between devices.

Bluetooth LE utilizes two methods to transmit data: broadcasting and connections. Broadcasting is used when a device chooses not to establish or is incapable of establishing a connection to another device. During broadcasting, a BLE device will intermittently send out advertising packets containing the required data about how to connect with the device. The data transmitted in these advertising packets are not encrypted, thus any kind of application data can be viewed by any device, making privacy nonexistent. Contrarily, connections are used when two or more devices need to exchange data. Connections begin with advertising packets to identify which device to connect. Once the devices have identified each other, they begin exchanging data packets. Connections serve the needs of private communications as they can implement BLE security features.

### 2.2. Bluetooth Low Energy Security

While security options are part of the BLE standard, the Bluetooth Special Interest Group (SIG) recommends but does not require security options to be implemented to allow flexibility for manufacturers. The manufacturer then decides whether security measures should be implemented or not. Within the host block, BLE devices can implement many security features, including association models, key generation, encryption, and random addressing. All these features can be used to ensure the security of the data transmitted during a connection to avoid threats, such as passive eavesdropping and MAC address fingerprinting.

Pairing is the most important security procedure in Bluetooth. Pairing is when authentication and key establishment take place between the two devices that connect. BLE defines two main pairing modes: Legacy Pairing and Secure Connections. BLE Legacy Pairing uses Secure Simple Pairing (SSP) of Bluetooth 2.1 (BR/EDR) but without the FIPS-approved Elliptic Curve Diffie-Hellman (ECDH) key exchange and HMAC-SHA-256 algorithms for pairing and message integrity [6]. Secure Connections (SC), introduced in Bluetooth 4.2, upgrades BLE Legacy Pairing to use ECDH with longer keys and provides data integrity. In this study, we focus on LE Legacy Pairing, which is the mode specified for Bluetooth versions 4.0 and 4.1.

LE Legacy Pairing uses a custom key exchange protocol, where the devices exchange a Temporary Key (TK) and use it to create a Short Term Key (STK) to be used as the encryption key for the communication. The pairing process is performed in three phases [10]:

Phase 1—Pairing Feature Exchange: The pairing process begins when the initiating device sends a pairing request to the other device. The two devices then exchange their capabilities and determine the association model they will use. There are three association models in BLE Legacy Pairing: Just Works, Passkey Entry, and Out of Band (OOB). Association models are used in the pairing process depending on the capabilities of each device to mutually authenticate devices attempting to establish communication. Passkey entry and Just Works association models generate a PIN code for both devices. For example, Just Works is designed for cases in which a device does not have the capabilities to enter a six-digit PIN code. Just Works has both devices agree on a six-digit number (0–999,999) that verifies the connection between the two. Passkey Entry requires one device to generate a PIN value, which is displayed on the device’s screen; the user will then match the value by entering it on the second device. It should be noted that there are certain input and output capabilities that each device must meet to use this model. OOB uses an alternative mechanism, such as Near Field Communication (NFC), to discover the devices and exchange or transfer cryptographic information to be used in the pairing process. These association models provide different levels of privacy and security. All data being exchanged during this phase is unencrypted, hence no protection against a passive eavesdropper is provided. OOB model may be an exception if the eavesdropper does not have the capability to capture the out of band channel or if the OOB traffic is encrypted. The Bluetooth 4.1 specification classifies the security properties of the association models into three categories: authenticated man-in-the-middle (MITM) protection, unauthenticated no MITM, and no security requirements. It states: “Authenticated man-in-the-middle (MITM) protection is obtained by using the passkey entry pairing method or may be obtained using the out of band pairing method. To ensure that Authenticated MITM Protection is generated, the selected Authentication Requirements option must have MITM protection specified” [10]. In BLE Secure Connections, a fourth association model called Numeric Comparison is added. In this model, both devices display a six-digit number on their displays and the user provides a “yes” response on each device if the numbers match. Numeric Comparison is the only model that provides protection against passive eavesdroppers.

Phase 2—Short Term Key (STK) Generation: In phase 2 of the LE Legacy Pairing, the devices generate and/or exchange the TK using one of the pairing methods described above. The two devices then exchange Confirm and Rand values to verify that they are both using the same TK. Once this has been determined, they use the TK, along with the Rand values, to create the STK. The STK is then used to establish an encrypted session to distribute the security keys, as explained in Phase 3. Note that STK is not exchanged between devices but generated on each device using the TK and a random value that is exchanged.

Phase 3—Transport Specific Key Distribution: This phase involves calculation and exchange of various keys. It is only performed on a link that is encrypted using the STK generated in Phase 2. The keys that are distributed in this phase are Long Term Key (LTK) for long term encryption, Encrypted Diversifier (EDIV), and Rand values to initially create and later identify the LTK; Identity Resolving Key (IRK) that allows a device to resolve a random address to the identity of its peer; and Connection Signature Resolving Key (CSRK), which allows the receiving device to authenticate the sender of the message. Bluetooth devices are assigned an address to communicate with other devices. However, there are multiple types of addresses used for varying levels of security. The Public Device Address, for example, is static, remaining the same throughout the device’s lifetime. The Random Device Address changes periodically, only allowing devices with permission to track the connection.

Although most security features are implemented in the host block, some mechanisms, such as Random Number Generation (RNG) and encryption algorithms, are executed in the controller to save CPU processing cycles and power. As a result, algorithms can be susceptible to poor implementation, which can jeopardize security.

### 2.3. Related Works

Bluetooth LE security has been explored in the past, especially when investigating privacy concerns during Bluetooth connections. In [11], the authors showed how BLE can be easily sniffed using affordable hardware, such as Ubertooth One. Ryan [12] used Ubertooth One in his work and exploited the many vulnerabilities of Bluetooth LE version 4.0. In his research, Ryan focused on BLE security, beginning with tracking the devices. He tracked the connections between devices by monitoring the Radio Frequency channels, determining the Bluetooth Access Address and ascertaining the hop increment (the hop increment determines how the two devices will change channels), which allows a connection to be tracked. Ryan also succeeded in bypassing the encryption by exploiting both Just Works and Passkey Entry using brute-force methods. Willingham, et al. [13] provided interesting results from a similar study to conducted by Ryan et al. [12]. They also used Ubertooth One to capture the packets sent during a Bluetooth LE connection. During the device analysis, the researchers observed security weaknesses in a keyboard, noting the methodology used to break the encryption scheme used. The other tested devices, a Fitbit and a smart watch, did not provide meaningful results, limiting the ubiquity of their conclusion that Bluetooth LE should not be used for sensitive data transfer. However, their work is nonetheless valuable to providing a precedence of weakness in at least one Bluetooth LE device. Hassan et al. [14] provided insight into the history of Bluetooth vulnerabilities and attacks on legacy Bluetooth. In this work, Hassan et al. provided a reference for many of the classic (often patched) Bluetooth vulnerabilities. However, their work on BLE-specific vulnerabilities is limited. Albazrqaoe et al. [15] provided an alternative method of tracking BLE devices. Chen et al. [16] and Lonzetta et al. [17] outlined many privacy issues present in the current BLE specifications, with a focus on Internet of Things devices. Both studies have provided foundational insight concerning devices’ security implementations. Ray et al. [18] discuss possible attacks on BLE field devices relevant for industrial automation and present a framework for defining and executing security attacks. They evaluate their framework on three BLE devices (SensorTag from Texas Instrument, Waspmote from Libelium, and a product prototype developed in-house) and conclude all three to be vulnerable. Das et al. [19] used static Bluetooth addresses to track devices (and their users), which compounds the already-existing privacy issues. The research discovered that many wearable fitness devices constantly broadcast advertising packets, which makes device tracking trivial. They also found that after a connection is established, the amount of user activity (e.g., exercise) correlates to the amount of data sent and received by the BLE devices. Consequently, a person can be actively identified within a small group based on their activity level (even with encrypted connections). As a remedy, Snader et al. [20] present CryptoCoP, an energy-efficient, content agnostic privacy and encryption protocol for IoT devices, including wearables.

Like the previously mentioned research, Hilts et al. [21] found that many fitness tracking devices do not utilize the address randomization available in the Bluetooth specifications. Additionally, they found that some devices do not employ encryption at all and all confidential and private information (e.g., personal information, email addresses, geolocation, and exercise habits) is sent in plaintext over the air. Zhang et al. [22] conducted attacks on four wearable devices, including Bong 3 HR, MiBand 2, TW64 and Mambo HR wristbands, and a Huawei Watch. Except for the smart watch, the devices were all vulnerable to various attacks, including replay, MITM, brute-force, and denial of service attacks. Similar work in [23] conducted attacks on the same types of devices, specifically on Fitbit Flex, by reverse-engineering the mobile applications that are used to connect and control the devices. They determined that Fitbit collects extraneous information about users, including the MAC addresses of nearby Fitbits. They also discovered that the Android application has access to step data with granularity down to a minute, but the user interface does not present this. Arias at al. [24] focused on IoT devices, specifically Google Nest Thermostat and the Nike+ Fuelband to show “how current industry practices of security as an afterthought or an add-on affect the resulting device and the potential consequences to the user’s security and privacy”.

Research on BLE security is not limited to wearables, sensors, or fitness devices. The literature also has analysis and attacks on other BLE devices showing the ubiquity of the issues with BLE security. Rose et al. [25] discussed how they hacked 12 smart locks out of 16. Cauquil [26] developed a MITM framework, called BTLEjuice, to conduct MITM attacks on smart padlocks, a robot, and a blood Glucose Monitoring System (GMS). Jasek et al. [27] demonstrated, through different attacks, such as MITM attacks, replay attacks, and reverse engineering of Bluetooth mobile applications, how they managed to hack and unlock different types of smart padlocks. Tan et al. [28] presented how they managed to hack bicycle locks used for bike sharing applications in Singapore. Gullberg [29] presented DoS attacks on BLE. Lounis et al. [30] evaluated the security of three BLE devices (bike lock, lightbulb, and a deadbolt) and showed how BLE makes Just Work not secure by introducing a new vulnerability for DoS attacks.

Throughout the research mentioned, there is a recurring pattern with Bluetooth Low Energy devices: privacy concerns with user data. In many cases, static Bluetooth Device Addresses allow tracking without any interference or defensive measures. Moreover, user information is often transmitted without the proper security implementations in place; this poses serious threats to user privacy, especially when considering the type of information being exchanged. Janesko [31] developed a framework by which Bluetooth Low Energy security mechanisms can be analyzed. In that work, Janesko describes the methods that can be used in BLE devices to secure a connection. Although Janesko’s framework proposes a robust auditing strategy, the challenges faced involved a lack of practical tools to practically analyze BLE devices. Janesko also provided a simplified, pragmatic listing and description of BLE security features for versions 4.0–4.2. In this direction, Issoufaly and Tournoux [32] used fingerprinting of BLE medium access control (MAC) addresses in wearables to track users via these addresses. They found that even though security and privacy features exist in BLE specifications, these features are rarely used. Robles-Cordero et al. [33] arrived at similar conclusions.

Our work contributes to the literature by adding security investigations of three 4.0/4.1 BLE-enabled heart rate monitoring/wearable sensing devices that are in the market today. The specific BLE manufacturers’ security implementations (the actual hardware/software manufacturers’ BLE implementations) of the three have not been previously investigated to the best of the authors’ knowledge. The work focuses on investigating whether the devices follow the recommendations by the Bluetooth SIG in terms of the implementation of the security mechanisms. We do not exploit the vulnerabilities in these devices but draw attention to the poor practices by manufacturers that put consumer security and privacy at risk. We propose the use of a standardized labeling mechanism to inform the consumer about the security of the Bluetooth devices they use.

## 3. Materials and Methods

**Figure 2:**
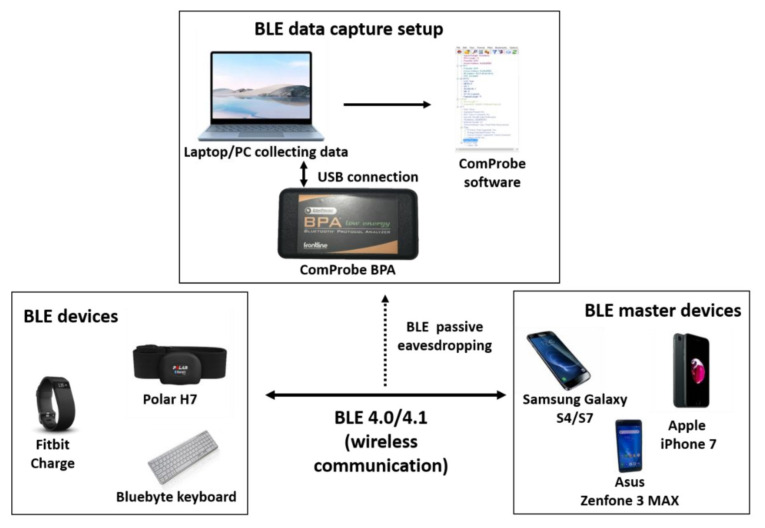
Experimental setup. The arrows in the figure indicate communication direction among devices/software. The dashed line indicates eavesdropping the communication channel.

We developed an experimental setup (as shown in Figure 2), with the main objective to investigate the traffic generated by the BLE when advertising, establishing connections, and sending data. We used the following devices for our experimental setup:

- ComProbe Bluetooth Protocol Analyser (BPA): This hardware device was used to capture wireless BLE traffic over a single BLE connection. At the same time, this device can capture all advertising packets in its vicinity. The ComProbe requires the use of ComProbe software, freely available at its website. Many studies on Bluetooth LE technology use the affordable Ubertooth One hardware for capturing BLE traffic. The authors had access to an Ubertooth One device and a ComProbe BPA during their research. Capturing traffic by ComProbe BPA was more reliable and convenient based on the authors’ experience, so they proceeded with ComProbe BPA in their study.
- Laptop/PC: These devices were used to connect the ComProbe device and to execute its software. They ran Windows 7 and 10, and the ComProbe BPA was connected via USB.
- BLE-enabled devices: We used two heart rate wearables and a BLE keyboard in our study. The wearables used were a Fitbit Charge (Bluetooth LE 4.1) and a Polar H7 Heart Rate Sensor (Bluetooth LE 4.0). The keyboard was a Bluebyte portable keyboard with Bluetooth LE 4.0. The Fitbit Charge is a wrist heart rate wearable, with official Apple iOS and Android apps, and it collects step count, distance covered, and calories burned (among other fitness data). The Fitbit Charge must use the official Fitbit app to work. The Polar H7 heart rate sensor straps onto the chest of a user and it transmits heart rate data. The Polar H7 has official apps for Android and iOS, but it can also be used without these official apps. Finally, the Bluebyte portable keyboard can connect to any Android/iOS and laptop/PC without the need of any app. Polar is a popular heart rate monitor brand with good rankings from consumer communities [34,35]. Even though Polar H9 is cited among the best HR trackers [34,35], the official website for Polar lists Bluetooth 4.0 to be the compatible Bluetooth version in their heart rate monitors [36]. The Polar H7 used in this study is an affordable device still in the market today [37], which implements the same Bluetooth version as its successor. Fitbit is a highly ranked smart watch/health tracker brand popular among consumers [38]. The Fitbit Charge 4 is an affordable Fitbit version available in the market [39,40]. The Bluebyte keyboard is also available in the market, advertised as a “Bluetooth V4.0 and 2.4G Wireless Multi-Device Keyboard” [41,42].
- BLE Master Bluetooth Devices: We used different smart phones and a PC, which served as BLE master devices. These were: an ASUS Zenfone Max 3, the Samsung Galaxy S4, a Samsung Galaxy S7, an Apple iPhone 7, and a HP Pavilion PC x360. All of these devices varied in Bluetooth version. For the smart phones, we downloaded and installed the software provided by the wearables’ manufacturers.

Once the software was installed on the devices, we captured data using the ComProbe network traffic analyzer. For the Fitbit Charge, we paired it with an iPhone 7, a Samsung Galaxy S4, and an ASUS Zenfone 3 Max. We paired the Polar H7 with the iPhone 7, Samsung Galaxy S4, Samsung Galaxy S7, and the ASUS Zenfone 3 Max. The Bluebyte keyboard was paired with the HP Pavilion x360, the iPhone 7, and the ASUS Zenfone 3 Max.

For the Polar H7 and the Fitbit Charge, we collected data for 1–2 min while walking to ensure that we had enough data collected through the BPA interface. We implemented a Java program that executed on the laptop/PC to format the data for readability. More specifically, the program reads in the packets from the text file exported from the ComProbe software, formats the results, and writes to a separate text file that includes only relevant information of the captured packets. The formatted data includes the frame number, timestamp, and heart rate value and stores it in another text file for viewing. The program also translates the raw hexadecimal data found in the exported file into both plaintext and binary format for additional readability and comparison. From here, we analyzed the data to observe the security measures that each device implemented.

## 4. Results

We observed varying levels of security implementation, showcasing inconsistencies in the device’s Bluetooth implementations. In some cases, devices were easily tracked, user data was compromised, or security measures were minimum (making attacks easier). Because the BLE devices attempt to be compatible when connecting, they may use the lowest set of Bluetooth security features available between both devices, thus, even though advance Bluetooth security features may be available to one device (among two paired/connected devices), these advanced features may not be used during a connection. This issue may create security and privacy issues.

### 4.1. Fitbit Charge

The Fitbit Charge implements the Passkey Entry association model, thus requiring a PIN to be matched by both devices before pairing. The tested device displayed the PIN on the screen of the Charge, and it was matched on a mobile device through a prompt on the Fitbit application. However, this PIN did not exactly match the specifications described by the Bluetooth Special Interest Group (SIG). The Charge utilized a four-digit PIN rather than the specified six-digits, which allowed a brute-force attack on the value to succeed extremely quickly. In a brute-force attack, the attacker attempts to compute all values possible for a given input (the PIN, in this case) until he or she finds a match. In addition, the Fitbit Charge did not use address randomization; thus, it could be easily tracked. However, the device did encrypt the content of its connection.

### 4.2. Polar H7 Heart Rate Sensor

We observed that the Polar H7 device did not stop broadcasting once it established a connection. In all captured data, the H7 continued to broadcast advertising packets intermittently. This also contributed to the ease of tracking for the device as it constantly broadcasted its device information even when connected to a BLE device.

When we inspected the collected packets more closely, the data showed that the device was leaking sensor data through the advertising packets, which contained heart rate data (in hexadecimal format). In addition, the advertising packets were always broadcasted/visible and were not encrypted. We discovered this leakage because the advertising packets contained this extra information, which had no header before the data itself, while the other data in the advertising packets had some header information identifying the data.

**Figure 3:**
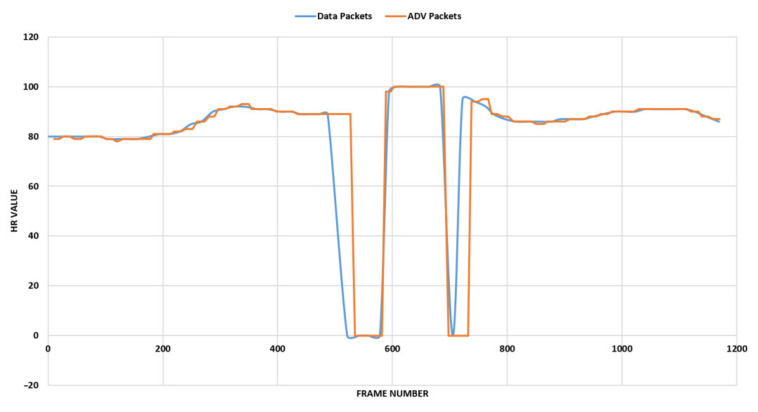
Polar H7 heart rate data/advertising packets.

We also found that the data packets sent by the H7 were not encrypted, which allowed us to correlate the heart rate data in the advertising packets to those sent as data packets. We found this relationship by looking at the heart rate data in a data packet, finding the closest (in time) captured advertising packet, and then matching the heart rate data. We conducted this procedure several times, and in all of them there was a matching of the heart rate data in both the data and advertising packets. With or without the Polar Beat mobile application, there was a possibility of user information leakage. In addition, we found that the H7 disconnects periodically from the user’s cell phone. We show the data collected in the data packets and the advertising packets in Figure 3.

### 4.3. Bluebyte Keyboard

When we analyzed the Bluebyte keyboard, it showed a level of security similar to the Polar H7. Despite the available security features in the BLE specification, the keyboard, upon analysis, has no data confidentiality or user authentication. This keyboard transmits a single character typed per data packet and the information is sent in plaintext. We analyzed the collected data and found data packets in sequence that spelled out the sentences we typed when capturing the network traffic. The packet even had flags that dictated if the “shift” key was pressed or if “Caps Lock” was on.

## 5. Discussion

With versions 4.0 and later of Bluetooth (including BLE), there have been many available measures in the standards to ensure confidentiality of user data, device authentication, and address randomization. However, the implementation of these security features is dependent on the individual manufacturer. In our analyzed devices, we found that they have vulnerabilities that could be exploited either to track users, collect potentially private information (e.g., health-related data), biometrics-related data, or, in the case of the keyboard that we studied, collect sensitive data, such as passwords or private identifiable data (e.g., social security numbers) due to the lack of encryption in the data packets. We summarize these results in Table 1.

In the case of the wearables that collect human physiological data, even though many devices are not medical grade (but fitness-related devices), they do not need to comply with security standards related to medical device security (at least in the U.S.), yet they still may create security vulnerabilities that could be exploited in the future by malicious actors.

Moreover, to facilitate access to hearing aids by the public, the U.S. government will drop the Food and Drug Administration (FDA) requirements to have prescription for certain kinds of these devices [43], allowing them to be bought as Over the Counter (OTC) hearing aids. If these hearing aids include BLE as one of its features (thus to serve as True Wireless Stereo devices, possibly collecting fitness data and voice data and serving as hearing aids at the same time) and these devices are not developed with security and privacy in mind, they will pose security and privacy threats to their users.

With technology advancing in other types of wearables, such as fitness trackers, wristbands, and smart watches, it is possible that a similar drop of certifications will happen in the future to collect fitness data as part of medical records. Thus, if these devices are not certified in their BLE security, they may pose violations to privacy laws in medical health records (e.g., Health Insurance Portability and Accountability Act (HIPPAA) laws in the U.S., or similar in other countries).

The lack of compliance in BLE security would be similar to the compliance in IoT privacy policies [44,45], in which a device states that it complies with certain practices/standards while working differently, thus potentially affecting the privacy, security, and safety of users. For example, stealing human physiological data in non-protected BLE connections could be exploited for replay attacks in biometric authentication methods based on wearable sensor data [46,47,48,49]. Biometric authentication systems may use wearables and wearable sensor data to authenticate users using single and multiple factors for both single and continuous authentication [50,51], and the leakage of BLE data could become a security/authentication issue.

Stolen physiological data could also be used in spoofing attacks to remote services, by which services could be exploited for financial gain (for example, an employee benefit service tricked to provide rewards for exercising [52]). Finally, unprotected BLE devices could be the subject of denial-of-service attacks or even be used to attack other devices or wireless networks [53,54].

## 6. Conclusion and Future Work

In this work, we conducted a study of the Bluetooth Low Energy security features of two heart rate consumer wearables, namely a Fitbit Charge and a Polar H7 wearable, and a BLE Bluebyte keyboard. We found that, even though security features are part of BLE standards, the devices we studied did not fully implement the security features of BLE, thus allowing users to be tracked (through MAC addresses in all the studied devices), and, in the case of the Polar H7 heart rate chest wearable and the Bluebyte keyboard, allowing the collection of data on a third-party device broadcasted from the wearables, thus violating the user’s privacy.

Given the vast amount of consumer wearables in the world, it is possible that many BLE devices bought by the public can be easily tracked, and user data that should be protected is easily accessible by hackers/rogue actors. While there is no certainty on a consumer device’s BLE security implementation until evaluated, in Table 2 we list questions that a consumer could ask himself/herself about the security/privacy of a BLE device before purchasing with the goal to evaluate the risk of BLE devices.

**Figure 4:**
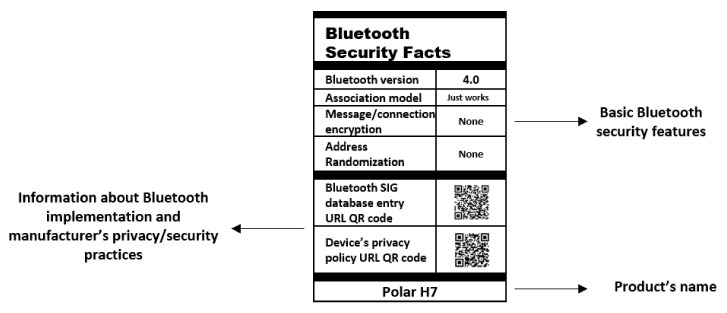
Design of a Bluetooth Security Facts Label (BSFL) for the Polar H7 wearable. In this example, the QR code for the device’s privacy policy encodes the URL of Polar’s website’s privacy notice.

We present in Figure 4 a prototype design of a Bluetooth Security Facts Label (BSFL) inspired by food nutrition packaging labels and similar research in privacy labels [55] to visually inform users about the Bluetooth/BLE security features of a device. In the future, Bluetooth SIG could require the incorporation of a BSFL label (or similar) in Bluetooth-enabled devices to identify the level of security implemented in the device.

More work is needed to provide an easy way for users to identify which consumer wearables (and other devices) implement security and privacy features of the BLE/Bluetooth standards and to educate the public on the threats from buying wireless devices that may not comply with such standards.

## Figures and Tables

**Table 1:** Summary of security issues found in BLE devices.

Device	BLE Version	Association Model	Address Randomization	Connection Encryption
Fitbit Charge wristband	4.1	Passkey entry	No	Yes
Polar H7	4.0	Just works	No	No
Bluebyte keyboard	4.0	Just works	No	No

**Table 2:** Questions to identify security practices when purchasing Bluetooth and BLE devices.

Question	Rationale
*Is the BLE device a “Qualified Bluetooth Device” listed on the Bluetooth Product Listing Database?*	All Bluetooth qualified devices are listed on the Bluetooth SIG Product Listing Database. Devices (or chips) not listed on this database are violating Bluetooth SIG rights/branding
*Does the product commercial packaging mention the Bluetooth/BLE versions that the device implements and/or any of its security features?*	No indication of BLE versions/security features could indicate absence of security
*Is the company that manufactures the BLE device a well-known company?*	Unknown/less-known companies could have practices that incur in technical debt, sacrificing security and user privacy
*Does the BLE device include a mobile app and/or mention a privacy policy associated with the device in its company website?*	The absence of privacy policies could indicate irregular/bad security and/or privacy practices. Companies could be liable if the privacy policy states that user/sensor data is protected but the BLE device leaks data
*Is the BLE device an FDA-approved device to diagnose, treat, or help manage a disease/health condition?*	FDA-approved devices must present, as part of their FDA certification process, a study of the security aspects of the device using FDA-recommended information security standards [1].
*Does the BLE device’s commercial packaging mention that the device complies with HIPPAA, Children’s Online Privacy Protection (COPPA), Gramm-Leach-Bliley Act (for financial privacy), or any other privacy law, such as the European Union’s General Data Protection Rules (GDPR)?*	Privacy laws, such as HIPPAA, COPPA, financial privacy, and GDPR, require privacy practices and protections for devices and systems involved in data collection for medical records, children’s data, financial data, and EU citizens’ data
